# Antibodies against peripheral nerve antigens in chronic inflammatory demyelinating polyradiculoneuropathy

**DOI:** 10.1038/s41598-017-14853-4

**Published:** 2017-10-31

**Authors:** Luis Querol, Ana M Siles, Roser Alba-Rovira, Agustín Jáuregui, Jérôme Devaux, Catherine Faivre-Sarrailh, Josefa Araque, Ricard Rojas-Garcia, Jordi Diaz-Manera, Elena Cortés-Vicente, Gisela Nogales-Gadea, Miquel Navas-Madroñal, Eduard Gallardo, Isabel Illa

**Affiliations:** 1 Neuromuscular Diseases Unit, Department of Neurology, Hospital de la Santa Creu i Sant Pau, Universitat Autònoma de Barcelona, Barcelona, Spain; 2 Centro para la Investigación Biomédica en Red en Enfermedades Raras, CIBERER, Madrid, Spain; 3 Fundación Favaloro, Buenos, Aires, Argentina; 4 Aix-Marseille Université, CNRS, CRN2M-UMR7286, Marseille, France; 5 Fundació Institut d’Investigació en Ciències de la Salut Germans Trias i Pujol, Badalona, Spain

## Abstract

Chronic inflammatory demyelinating polyradiculoneuropathy (CIDP) is a heterogeneous disease in which diverse autoantibodies have been described but systematic screening has never been performed. Detection of CIDP-specific antibodies may be clinically useful. We developed a screening protocol to uncover novel reactivities in CIDP. Sixty-five CIDP patients and 28 controls were included in our study. Three patients (4.6%) had antibodies against neurofascin 155, four (6.2%) against contactin-1 and one (1.5%) against the contactin-1/contactin-associated protein-1 complex. Eleven (18.6%) patients showed anti-ganglioside antibodies, and one (1.6%) antibodies against peripheral myelin protein 2. No antibodies against myelin protein zero, contactin-2/contactin-associated protein-2 complex, neuronal cell adhesion molecule, gliomedin or the voltage-gated sodium channel were detected. In IgG experiments, three patients (5.3%) showed a weak reactivity against motor neurons; 14 (24.6%) reacted against DRG neurons, four of them strongly (7.0%), and seven (12.3%) reacted against Schwann cells, three of them strongly (5.3%). In IgM experiments, six patients (10.7%) reacted against DRG neurons, while three (5.4%) reacted against Schwann cells. However, results were not statistically significant when compared to controls. Immunoprecipitation experiments identified CD9 and L1CAM as potential antigens, but reactivity could not be confirmed with cell-based assays. In summary, we describe a diverse autoantibody repertoire in CIDP patients, reinforcing the hypothesis of CIDP’s pathophysiological heterogeneity.

## Introduction

Chronic inflammatory demyelinating polyradiculoneuropathy (CIDP) is a disabling disease with a pathogenesis that remains largely unknown^1^. CIDP response to immune therapies and scarce experimental evidence on passive transfer animal models suggest that humoral factors play a role in its pathogenesis^2^. CIDP diagnosis is based on clinical and electrophysiological criteria^3^ that allow the inclusion of a broad spectrum of patients within CIDP, including typical and atypical variants. This heterogeneity has hindered the description of disease-specific biomarkers, despite intensive research efforts^4^.

The response of CIDP patients to intravenous immunoglobulin (IVIg) and plasma exchange (PlEx) suggests that humoral factors are involved in its pathogenesis. The search of autoantibodies has been the most important laboratory research topic in CIDP. Initial focus was placed on myelin antigens. Classical studies, using diverse techniques, detected higher frequencies of antibodies against myelin protein zero (MPZ), peripheral myelin protein 2 (PMP2) or peripheral myelin protein 22 (PMP22)^5–10^. However, meaningful clinical-immunological correlations with those antigens were not established. CIDP patients harboring antibodies against LM1-containing ganglioside complexes, present more frequently with ataxia, although these results await replication in independent cohorts^11,12^. We and others have recently detected antibodies targeting node of Ranvier proteins such as gliomedin, neuronal cell adhesion molecule (NrCAM), neurofascin 140 (NF140), neurofascin 186 (NF186); and paranode of Ranvier; contactin-1 (CNTN1), contactin-associated protein 1 (CASPR1) and neurofascin 155 (NF155)^13–17^. Our group described that patients harboring antibodies against CNTN1 and NF155 of the immunoglobulin G4 (IgG4) isotype present with specific clinical features^15,18^. Studies by other groups have validated these clinical-immunological correlations and refined the clinical phenotypes and paraclinical features associated to these autoantibodies^19–23^. *In vitro* and *in vivo* models of anti-CNTN1 IgG4 passive-transfer, demonstrate that anti-CNTN1 antibodies are pathogenic, strengthening the idea that CIDP is an autoantibody-mediated disease^24^.

Considering that CIDP-specific autoantibodies are not detected in the majority of CIDP patients we developed this study to: (1) systematically screen for immunoglobulin G (IgG) and immunoglobulin M (IgM) autoantibodies against previously described antigens and peripheral nerve components, (2) identify the molecular targets of the immune response in those patients reacting against peripheral nerve components in which the target antigens were unknown and (3) to establish clinical-immunological correlations.

## Results

Sixty-five patients fulfilling criteria for CIDP were identified and included in the study. Twenty-nine of them (29/65; 44.6%) were women (mean age 64.0 ± 17.3 years) while 36 of them (36/65; 55.4%) were men (mean age 61.3 ± 15.3 years). Forty-four patients (67.7%) presented with a typical CIDP while 21 presented with an atypical CIDP (32.3%) (Supplementary Table S1).

**Figure 1:**
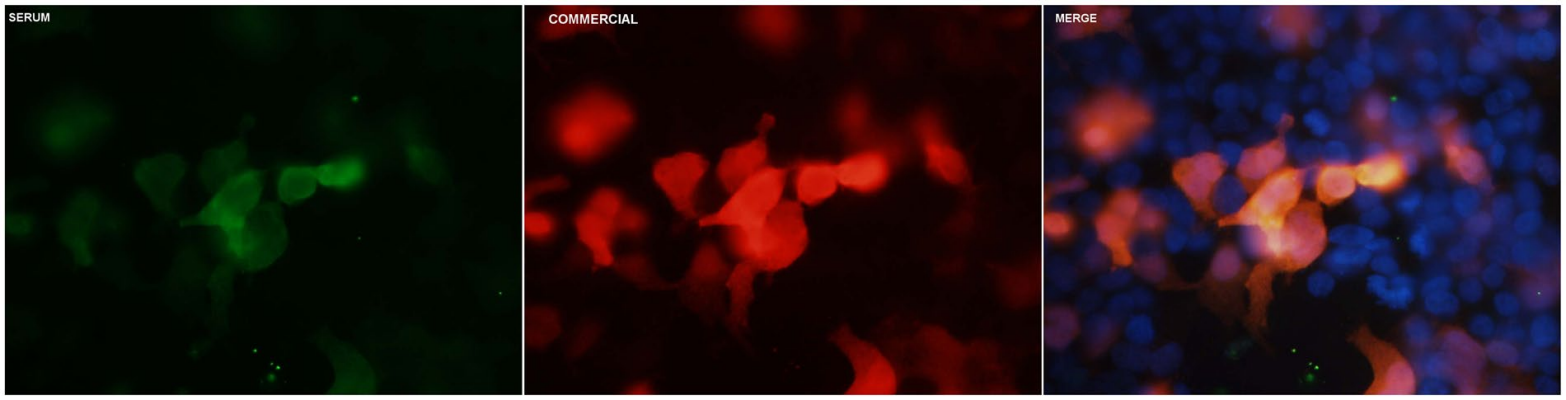
Positive PMP2 ICC. HEK293 cells were transfected with a mammalian expression vector encoding PMP2 with the use of Lipofectamine 2000 (Invitrogen, CA, USA) and ICC was performed as described in Supplementary Table S3. Patient’s 22 sera positivity can be appreciated in green, commercial antibody staining in red and a merged picture of both can be found above with nuclei stained in blue.

Eleven patients (11/59; 18.6%) showed anti-ganglioside antibody reactivity by enzyme-linked immunosorbent assay (ELISA). Four of them (6.7%) showed high anti-GM1 IgM antibody titers (two of them with anti-GD1b IgM, with or without additional reactivities) (Table 1). Four patients reacted against CNTN1 (4/65; 6.2%), one against the CNTN1/CASPR1 complex (1/65; 1.5%) and three against NF155 (3/65; 4.6%). All of them except two (one CNTN1 and one NF155 positive) were previously described elsewhere^15,18^. Only one patient (1/62; 1.6%) reacted against PMP2 (Fig. 1). CSF from this patient also showed IgG reactivity against PMP2-transfected human embryonic kidney (HEK) cells. None of our CIDP patients reacted against any of the other candidate antigens (MPZ, NrCAM, contactin-2 (CNTN2)/contactin-associated protein 2 (CASPR2) complex, gliomedin and voltage-gated sodium channel subunits B1 (NavB1) and B2 (NavB2)). For further detailed results see Supplementary Table S2.

**Table 1:** CIDP patients harbouring anti-ganglioside antibodies.

Patient ID	Results
7	sulfatides IgM 1/31356
	sulfatides IgG 1/832
22	IgM aGM1 1/500
24	sulfatides IgM 1/592
31	IgG GM1 1/6160
	IgM GM1 1/2314
	IgM aGM1 1/1442
	IgM GD1b1/500
32	IgG aGM1 1/580
36	IgM GM1 1/2154
39	IgG aGM1 1/528
	IgG GD1a 1/528
	IgM aGM1 1/2559
	sulfatides IgM 1/3245
	sulfatides IgG 1/831
55	sulfatides IgM 1/800
	IgM aGM1 1/500
56	IgG aGM1 1/1000
	IgM GM1 1/500
64	IgM GM1 > 1/12500
	IgM aGM1 > 1/12500
	IgM GD1b > 1/12500
65	IgM GM1 1/7829
	IgM aGM1 1/2359
	IgM GD1b 1/2868

Fifty-nine patients were screened using our institution’s anti-ganglioside ELISA diagnostic technique, further confirmed by TLC.

Immunocytochemistry (ICC) experiments with dorsal root ganglion (DRG) and motor neurons and Schwann cells (human undifferentiated Schwann cell line) were used to identify novel reactivities against neural components. Patients with anti-CNTN1, anti-CNTN1/CASPR1, who show strong reactivity against any type of neuron, and anti-NF155 antibodies, who do not react against neurons, were excluded from these analyses. As a result, a maximum of 57 patients were screened for novel reactivities. During the course of our experiments we ran out of sera from one of the patients, leaving a total of 56 patients in IgM experiments.

**Figure 2:**
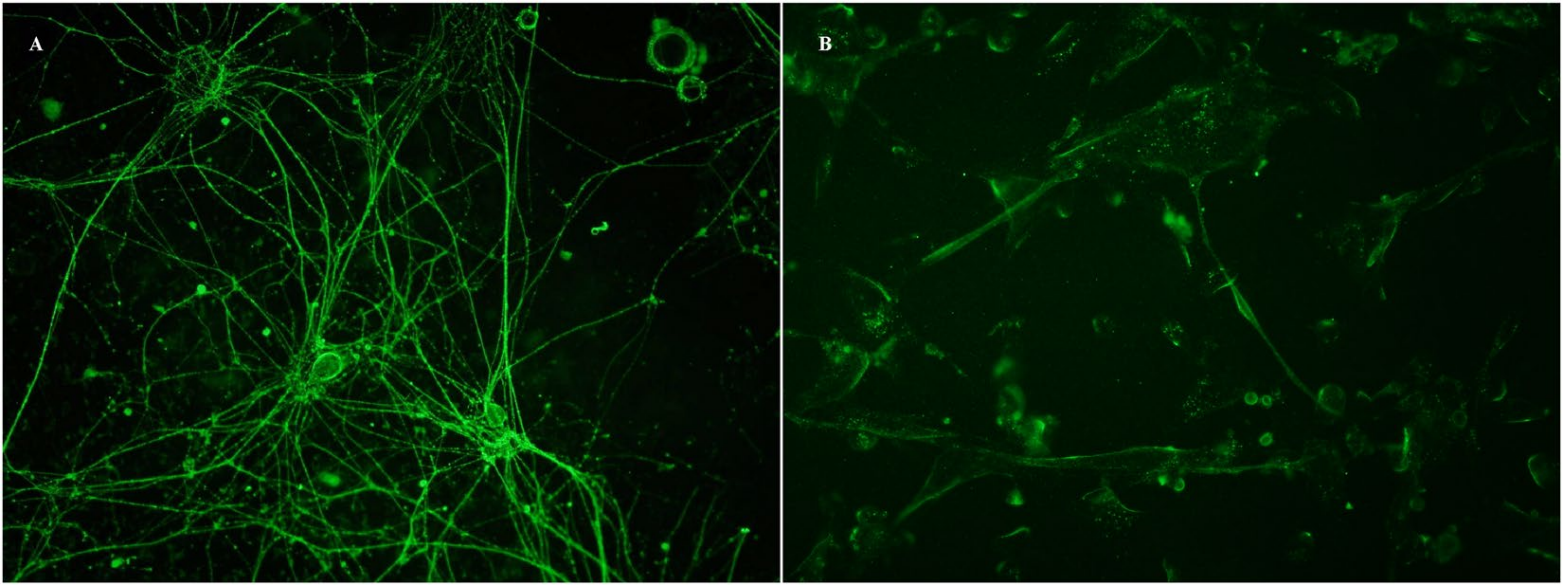
IgG positivity in DRG neurons and Schwann cells. Patients’ sera were tested by IgG and IgM ICC experiments with live DRG neurons and Schwann cells. Strong staining with the use of an anti-human IgG Alexa Fluor 488 antibody (Thermo Fisher Scientific, MA, USA) can be appreciated for DRG neurons (**A**) and Schwann cells (**B**).

In IgG experiments, three patients (3/57; 5.3%) showed only mild IgG staining in motor neuron ICC and therefore were not further used for immunoprecipitation (IP) experiments. Fourteen patients (14/57; 24.6%) presented with IgG reactivity against DRG neurons. Of those, only four (4/57; 7.0%) showed moderate to intense reactivity against DRG (Fig. 2A). Sera from three of these patients showing moderate or strong reactivity and a normal control were used to try to identify the target antigens using DRG neurons as the IP substrate. No relevant antigens were identified in comparison to the control serum. Finally, we used a commercial human undifferentiated Schwann cell line to screen for antibodies against Schwann cells. Seven patients (7/57; 12.3%) showed IgG reactivity towards the human Schwann cell line, although only three (3/57; 5.3%) showed consistent moderate to high intensity staining (Fig. 2B). Interestingly, the only patient showing antibodies against PMP2 showed weak reactivity against human Schwann cells. Sera from these three patients strongly reacting against human Schwann cells and one control serum were used for IP experiments, but mass spectrometry analysis did not reveal any relevant antigen in CIDP patients when compared to the control individual. Two patients with weak reactivity against Schwann cells also showed weak staining in DRG neurons ICC. Another patient, highly reactive against Schwann cells also showed weak reactivity against DRG neurons. No cross-reactivity was found between motor neurons and DRG neurons or Schwann cells (see Table 2). Additionally, statistical comparison between ICC results from patients and controls did not provide evidence of differentially relevant IgG staining in DRG neurons (p = 0.6793) or Schwann cells (p = 0.5476) (Table 3).

**Table 2:** CIDP patients with moderate to strong reactivity in ICC experiments.

Patient ID	Motor neurons IgG	Schwann cells IgG	Schwann cells IgM	Drg neurons IgG	Drg neurons IgM	PMP2ICC
12	0	0	0	2	0	0
18	0	0	0	1	2	ND
22	0	0	0	0	0	3
23	0	3	1	1	0	0
33	0	0	ND	3	ND	0
48	0	0	0	2	0	0
50	0	3	1	0	0	0
56	0	2	0	0	0	0
58	0	0	2	0	0	0
64	0	1	0	2	0	0

**Table 3:** Statistical analysis of DRG neurons and Schwann cells ICC in CIDP patients and healthy controls.

	Controls	Patients	P value
Schwann cells IgG ICC	0/28 (0%)	3/57 (5.3%)	0.5476
Schwann cells IgM ICC	0/28 (0%)	1/56 (1.8%)	1
DRG neurons IgG ICC	3/28 (10.7%)	4/57 (7.0%)	0.6793
DRG neurons IgM ICC	2/28 (7.1%)	1/56 (1.8%)	0.2565

Moderate to strong fluorescence intensity scores, including scores two and three, and other stainings, featuring scores zero and one, from DRG neurons and Schwann cells IgG and IgM ICC experiments from patients and controls were analyzed using contingency analysis with the application of a two-tailed Fisher’s exact test, accepting an alpha-level <0.05 to determine significance.

In IgM experiments, six patients (6/56; 10.7%) showed reactivity against DRG neurons, while three (3/56; 5.4%) reacted against a human Schwann cell line. Only one patient (1/56; 1.8%) per each group presented with moderate (score two out of three) intensity IgM staining (Table 2). As with IgG experiments, no cross-reactivity was either appreciated between both cell types. IgM IP analysis were not performed in this study, due to the staining being exclusively moderate and poorly significant, as only one in 56 (1.8%) patients featured it in both cell types. As with IgG experiments, statistical comparison between IgM ICC results from patients and controls showed no statistically significant differences between both groups in DRG neurons (p = 0.2565) or Schwann cells (p = 1) (Table 3).

Whole nerve lysate from rat sciatic nerve and human cauda equina served as substrate for IP experiments with pooled sera from five typical CIDP cases. L1 cell adhesion molecule (L1CAM), a protein of the neurofascin family, was identified as a potential candidate antigen in the whole-nerve sciatic nerve lysate IP experiment but confirmatory ICC tests with L1CAM-transfected HEK cells did not confirm reactivity in any of the typical CIDP cases. Cluster of differentiation 9 (CD9), a protein of the tetraspanin family present in the paranode of Ranvier, was identified as a potential candidate antigen using human cauda equina as the substrate for IP studies with typical CIDP cases but, again, this was not confirmed in ICC experiments using CD9-transfected HEK cells.

No specific clinical features were associated with reactivity against each neural cell. The two patients with IgM antibodies against GM1 plus GD1b presented with an asymmetric sensory-motor onset in the upper limbs compatible with Lewis-Sumner CIDP variant, although later in the disease course progressed towards a bilateral, symmetric polyneuropathy. The other two patients with anti-GM1 antibodies presented with a typical CIDP variant. Interestingly, three of the anti-GM1 positive patients showed normal sensory nerve conductions despite severe sensory involvement, suggesting preganglionar damage. The fourth patient showed sensory-motor involvement in the EMG. Finally, the only patient reacting against PMP2 presented with a typical CIDP phenotype.

## Discussion

Our study describes a comprehensive autoantibody screening approach to identify clinically relevant antigens in CIDP, and provides experimental evidence of the immunopathological diversity in patients fulfilling CIDP diagnostic criteria. A subset of seronegative CIDP patients reacted against neural structures and gangliosides. However, IP experiments in patients reacting against neural cells did not reveal novel antigens, and frequencies of reactivity against neural cells did not differ from normal controls. We did not identify patients harboring antibodies against node/paranode of Ranvier proteins other than CNTN1 or NF155.

Aside from the inclusion of a few new CIDP patients in comparison to previous studies^15,18^, the novelty of our work relies on describing one new CNTN1 positive patient, one new NF155 positive patient, one patient positive for PMP2 and 11 CIDP patients with anti-ganglioside antibodies. Additionally, we have tested candidate antigens described in the bibliography and performed systematic antigen screening in rat dorsal root ganglion neurons, rat motor neurons and a human undifferentiated Schwann cell line. Systematic screening of serum reactivity against the two neuronal components of peripheral nerves, DRG and motor neurons, has never been performed before. In fact, our previous studies used hippocampal neurons, rat teased nerve fibers and rat brain sections to screen reactivity^15,18^. Therefore, although a partial sample overlap must be acknowledged, the different substrates used in our methods grant novelty and relevance to our results.

The search for diagnostic biomarkers in CIDP has been a research topic for decades^2^. Most patients with CIDP show an excellent response to PlEx and IVIg^25,26^. Response to IVIg follows time dynamics that strongly suggest direct competition of the therapeutic immunogloblulins with pathogenic autoantibodies^27^. The presence of IgG, IgM and complement deposits has been described in chronic inflammatory neuropathies for almost 40 years^28^ and confirmatory studies that passive transfer of IgG from CIDP patients led to demyelinating nerve pathology were performed 17 years ago^29^. However, the target antigens for most CIDP patients remain elusive. Considering the demyelinating nature of CIDP, the neuritogenic potential of myelin proteins and their discovery in some demyelinating hereditary neuropathies, myelin proteins were extensively studied as potential antigens in CIDP. MPZ, PMP2, PMP22 and connexin-31 among others were tested as possible candidate antigens, but clinically relevant autoantibodies could not be established^5,7,10,30–32^. We tried to replicate the results with two of those antigens (MPZ and PMP2) and found only one patient with antibodies against PMP2, both in serum and CSF, precluding further clinical correlations.

Gangliosides and ganglioside complexes are well-established targets of humoral responses and provide useful clinical-immunological correlations in diverse inflammatory neuropathies^33^. Considering this, anti-ganglioside antibodies have also been studied in CIDP and other chronic inflammatory neuropathies^34,35^. Diverse CIDP-associated anti-ganglioside antibodies have been reported but, so far, antibodies targeting LM1-containing ganglioside complexes are the only ones associated to specific clinical features such as ataxia^12^. Anti-ganglioside antibodies were systematically tested in our patients by ELISA and further confirmed with thin-layer chromatography (TLC). Four patients showed IgM antibodies against GM1. Two of them, who also had GD1b IgM antibodies, presented with the Lewis-Sumner variant of CIDP. Previous reports describing the clinical and immunological features of Lewis-Sumner did not find any association with GM1 antibodies or proposed them as a differential feature compared to multifocal motor neuropathy (MMN)^36,37^. Description of clinical-immunological correlations in larger cohorts could clarify if there is a subset of CIDP patients with well-established anti-ganglioside antibody reactivities. Seven patients showed low anti-ganglioside/sulfatide antibody titers, probably without clinical relevance.

Our group and others have used candidate-antigen and unbiased approaches to study proteins of the node of Ranvier as potential targets of the immune response in CIDP^13–15,17^. Devaux’s and Yuki’s groups described antibodies against nodal proteins in patients with CIDP and Guillain-Barré Syndrome (GBS), including IgG and IgM antibodies against neurofascin, gliomedin, CNTN-1 and NrCAM^13^. Later on, Meinl’s group described antibodies against neurofascin in very specific subsets of CIDP (and GBS) patients^14^. Our group published that a small subset of CIDP patients reacted strongly against neurons and that CNTN1 and the CNTN1/CASPR1 complex were the antigens in those patients^15^. Interestingly, these patients showed specific clinical features and poor response to IVIg, likely due to the IgG4 nature of the autoantibodies. We then described that some patients reacting against paranodal structures, harbored IgG4 anti-NF155 antibodies. These patients also showed poor response to IVIg and very specific clinical features^18^. Ever since, other groups have confirmed in independent cohorts the presence of anti-CNTN1 and anti-NF155 antibodies in very specific and infrequent CIDP^19–22^, and passive transfer of anti-CNTN1 IgG4 antibodies has demonstrated to be pathogenic^24^. The presence of antibodies against nodal neurofascins (NF140 and NF186) has been recently associated to specific clinical features, including an interesting association with nephrotic syndrome^16^. However, other nodal antigens such as NrCAM or gliomedin have not been replicated yet. We tried to identify CIDP patients with antibodies against those nodal antigens and the CNTN2/CASPR2 complex and the two subunits of the nodal sodium channel but failed to identify any positive patients in contrast to previous studies. The low frequency of these antibodies may account for some of these disparities; larger cohorts in which systematic autoantibody screening is performed should be studied.

For seronegative CIDP patients we developed a screening approach to identify IgG and IgM antibodies using the three main cellular components of the peripheral nerve: motor neurons, DRG neurons and Schwann cells. A significant number of patients (22/57; 38.6%), showed reactivity against any of the three relevant cellular components (a similar proportion to that found in other studies)^13,38^. Most of them showed exclusive reactivity against a single cell type, but a few reacted against two different cell types. Nine patients reacted strongly against DRG or Schwann cells and deserved inclusion in IP experiments. Presence of high concentrations of immunoglobulins in sera after IVIg treatment, might have contributed to unspecific staining, particularly of the IgG subtype, in our patients’ ICC experiments. In order to avoid this bias, as stated in the methods section, patients’ and controls’ ICC results were compared according to two separate categories: moderate to strong stainings, (including scores two and three respectively) and other stainings (including scores one and zero).The latter category being encompassed by negative samples (0) and samples with irrelevant background staining (1), including those that might have featured an unspecific staining as a consequence of higher immunoglobulin titers after IVIg treatment. Since only samples showing moderate or strong reactivities against peripheral nerve cells were used for IP experiments, IVIg treatment cannot be considered a confounding variable in our study. Frequencies of sera reacting against neural structures did not reach statistical significance, likely due to the small number of controls and to a relatively high proportion of controls showing moderate unspecific staining, but it could be, as has been suggested for anti-ganglioside antibodies, that naturally occurring antibodies targeting nerve structures, are frequent in healthy population^39^. No relevant antigens were identified from the mass spectrometry analysis of the precipitated samples.

Finally we attempted whole nerve IP to discover antigens in typical CIDP patients and found two potential candidates: CD9, a tetraspanin present in the paranode^40^, and L1CAM, a protein of the neurofascin family. Unfortunately, we failed to confirm autoantibodies against these two antigens in CIDP. The group of Yuki also screened antibodies against CD9 and other tetraspanins and did not find such autoantibodies in CIDP either^41^.

Although the use of several antigens of murine origin (mammalian expression vectors encoding nodal proteins and neuronal primary cultures) may be considered as a limitation of our study, their utility in previous works and in autoimmune encephalitis encouraged us to pursue novel antigen screening in murine models. Inter-species antigen variability, although minor in terms of sequence homology, may however be very relevant for autoantibody screening purposes. This happens with other nerve antigens such as myelin-associated glycoprotein in polyneuropathy associated with monoclonal gammopathy or NF155 and should be taken into account in future studies. The use of human neural cells obtained from induced pluripotent stem cells could overcome these technical difficulties^42^. A very interesting study identified a subset of patients with inflammatory neuropathies reacting against human Schwann cells and, thus, set the proof of principle that ICC over Schwann cells in isolation may also be useful for autoantibody screening purposes^38^. One important issue regarding our model is that it uses murine cells or undifferentiated human Schwann cells. The study by Kwa and coworkers used human Schwann cells obtained from sural nerve biopsies and amputation material. The scarcity of this type of sample precludes routine use of these two cell sources. Exploring novel ways of obtaining differentiated human neural cells would therefore help refining these screening techniques.

Another limitation is that, although our screening approach is valid for any type of antigen, identification with IP and mass spectrometry is only useful for protein antigens and, thus, antigens of lipidic or glucidic nature cannot be identified. Finally, identification of relevant protein antigens with IP and mass spectrometry relies on completeness and accuracy of existing protein databases. Although murine and human protein databases cover a very significant proportion of the proteome, their completeness, accuracy and detail may be insufficient for novel antigens that may have not been studied in any other disease or model, and thus it is difficult to assign relevance to the identified antigens.

CIDP is defined based on broad clinical and electrophysiological criteria that successfully identify patients that may benefit from immunomodulatory therapy. However, the inclusion of patients with diverse clinical features into the same diagnostic category leads to clinical, electrophysiological, radiological and pathological heterogeneity that, in the end, interferes with translational research aimed to identify clinically meaningful biomarkers and, among them, autoantibodies. Our study provides the most comprehensive attempt to discover novel antigenic reactivities in CIDP and shows that the pattern of IgG and IgM reactivity of CIDP patients is heterogeneous and targets diverse nerve proteins and structures, further proving the difficulty in the identification of new biomarkers in this context. Our results may therefore help to understand the disparity in previous reports on autoantibodies in CIDP and supports the idea that larger CIDP registries that attempt to collect and homogenize clinical and biomarker information are very much needed.

## Materials and Methods

### Patients, informed consent and protocol approvals

Sixty-five consecutive patients fulfilling European Federation of Neurological Societies/Peripheral Nerve Society (EFNS/PNS) diagnostic criteria for CIDP and followed in Hospital de la Santa Creu i Sant Pau were included in this study. Twenty-eight healthy controls were additionally included in our experiments. Serum samples were obtained at inclusion in the study and stored at −80 °C until needed. Written informed consents were obtained from all subjects according to the Declaration of Helsinki. Participation in the study was conducted under a protocol approved by the Ethics Committee of the Hospital de la Santa Creu i Sant Pau. All experiments were performed in accordance with the relevant guidelines and regulations.

In regard to experiments involving rats, all experimental procedures were approved by our institution’s Service of Animal Experimentation at CSIC-ICCC (Institut Català de Ciències Cardiovasculars). All experiments were performed in accordance with due guidelines and regulations.

### Protocol overview

The autoantibody screening in our patients was designed as a multi-step process (Supplementary Fig. S1). First, patients’ sera were tested for antibodies against gangliosides and against previously described antigens from myelin (MPZ, PMP2) or node of Ranvier; CNTN1, NF155, NrCAM, gliomedin, the CNTN1/CASPR1 and the CNTN2/CASPR2 complexes. Antibodies against the two subunits of the main sodium channel present at the node of Ranvier; NavB1 and NavB2 were also tested. Patients showing positivity towards CNTN1, CNTN1/CASPR1 or anti-NF155 (n = 8), all of them except two previously described elsewhere^15,18,43^, were excluded from statistical analysis in screening experiments, and were only used in such experiments as controls. These patients were neither tested in further IP analysis. Only patients harboring no reactivity against previously described antigens (n = 57) and healthy volunteers serving as negative controls (n = 28) were included in autoantibody screening experiments analysis.

Primary cultures of rat DRG and motor neurons, and a human, undifferentiated, Schwann cell line (ScienCell, CA, USA) were used to screen reactivity against peripheral nerve cells. Patients showing autoantibodies against any of these nerve structures were used for antigen discovery with immunoprecipitation. Sera of five typical CIDP cases were pooled and incubated in IP experiments with whole nerve lysate obtained from rat sciatic nerve and human cauda equina obtained from the IDIBAPS (Institut d’investigacions Biomèdiques August Pi i Sunyer) neural-tissue bank. Finally, if any candidate antigen was detected in any of the IP experiments, confirmatory experiments with transfected HEK cells encoding the protein of interest, followed.

### Cell cultures

DRG and motor neurons were isolated and cultured following published protocols with minor modifications^44,45^. Briefly, DRG and spinal cords were dissected from E16 rat embryos and dissociated to a cell suspension in neurobasal medium (Gibco BRL, NY, USA) supplemented with B27 (Gibco BRL, NY, USA), Glutamax (Gibco BRL, NY, USA) and nerve growth factor (NGF) (Invitrogen, CA, USA). After 24 hours plated in glass-coverslips, cytosine arabinoside (ARA-C) (Sigma, MO, USA) and fluorouracil (5-FU) (Sigma MO, USA) were added to the medium to remove fibroblasts in DRG neurons cultures. In both cultures, medium was replaced every other day until reaching motor or DRG neuron full growth.

A commercial human Schwann cell line (ScienCell, CA, USA) was cultured following manufacturer’s instructions and plated onto glass coverslips until 70–80% confluence was reached.

### DRG neuron, motor neuron and Schwann cell immunocytochemistry

Live DRG neurons, Schwann cells and motor neurons were incubated with patients’ sera diluted in culture medium. Cells were then fixed with 4% paraformaldehyde (Affymetrix Inc, CA, USA) and incubated with secondary goat anti-human IgG/IgM Alexa Fluor 488 antibody (both by Thermo Fisher Scientific, MA, USA). DRG neurons and Schwann cells were tested against IgG and IgM reactivities, both in patients and controls. Due to the limiting scarcity of rat motor neurons extraction, only patients were tested against IgG in these experiments. Finally, coverslips were mounted with Vectashield with DAPI (Vector Laboratories, CA, USA). Fluorescence signal intensity was scored in a 0–3 scale by two independent researchers. Relevant images were obtained with the use of an Olympus BX51 Fluorescence Microscope (Olympus Corporation, Japan) and processed with ImageJ (U. S. National Institutes of Health, MD, USA).

### Immunoprecipitation

Sera showing moderate or strong reactivity against peripheral nerve cells were used for IP experiments using the same cell as IP substrate. Protein A and G agarose beads (Invitrogen, CA, USA) were used to isolate sera IgG bound overnight to the antigens in a cell culture extract. Precipitated proteins were detached from the agarose beads with Laemmli buffer (Bio-Rad, CA, USA) with 5% b-mercaptoethanol (Merck, Germany) and separated by electrophoresis. Bands appearing in patients’ IP but not in control’s were analyzed by mass spectrometry. Proteins were selected as candidate antigens when they fulfilled any of these criteria: protein score > 100, peptide sequence coverage >5% or two or more peptides identified with the absence of the same criteria in the control sample.

A subset of five patients with typical CIDP that did not react against nerve cells was used for IP experiments using rat whole nerve lysate or human cauda equina as the IP substrate following the exact same protocol to analyze precipitated proteins.

### Anti-ganglioside antibody screening

Fifty-nine patients were screened for the presence of anti-ganglioside antibodies with our institution’s diagnostic protocol using ELISA^46^ as the general detection method and TLC^47^ for confirmatory experiments. Anti-ganglioside antibodies were considered positive at a 1:500 titer. The remaining six CIDP patients in our cohort could not be analyzed due to sample scarcity.

### HEK cell transfection and ICC

HEK293 cells were transfected with Lipofectamine 2000 (Invitrogen, CA, USA) with mammalian expression vectors encoding human MPZ, PMP2, gliomedin, CNTN1, CASPR1, CNTN2, CASPR2, NF155, CD9 and L1CAM, and murine NrCAM and NavB1 and NavB2. Cells were then fixed with 4% paraformaldehyde (Affymetrix Inc, CA, USA), permeabilized (if needed) with 0.3% TritonX-100 (Sigma, MO, USA) and blocked. ICC experiments were performed using patients’ sera and appropriate primary and secondary antibodies (Supplementary Table S3).

### Statistical analysis

Fluorescence intensity scores from DRG neurons and Schwann cells IgG and IgM ICC experiments from patients and controls were analyzed by Stata v.13.1 (StataCorp LP, Texas, USA) using contingency analysis with the application of a two-tailed Fisher’s exact test, accepting an alpha-level <0.05 to determine significance. Patients’ and controls’ ICC results in each condition were compared according to two separate categories: moderate to strong staining, including scores two and three, and other stainings. Scores two and three were considered positive, while score one was interpreted as unspecific background staining.

### Data availability

All data generated or analysed during this study are included in this published article (and its Supplementary Information files).

## Electronic supplementary material

Supplementary Information

## References

[1] Mathey EK (2015). Chronic inflammatory demyelinating polyradiculoneuropathy: from pathology to phenotype. J. Neurol. Neurosurg. Psychiatry.

[2] Querol L, Devaux J, Rojas-Garcia R, Illa I (2017). Autoantibodies in chronic inflammatory neuropathies: diagnostic and therapeutic implications. Nat. Rev. Neurol..

[3] Van den Bergh PYK (2010). European Federation of Neurological Societies/Peripheral Nerve Society guideline on management of chronic inflammatory demyelinating polyradiculoneuropathy: report of a joint task force of the European Federation of Neurological Societies and the Peripheral Nerve Society. Eur. J. Neurol..

[4] Brannagan TH (2011). Current diagnosis of CIDP: the need for biomarkers. J. Peripher. Nerv. Syst..

[5] Yan WX, Archelos JJ, Hartung H-P, Pollard JD (2001). P0 protein is a target antigen in chronic inflammatory demyelinating polyradiculoneuropathy. Ann. Neurol..

[6] Gabriel CM, Gregson NA, Hughes RA (2000). Anti-PMP22 antibodies in patients with inflammatory neuropathy. J. Neuroimmunol..

[7] Kwa MS, van Schaik IN, Brand A, Baas F, Vermeulen M (2001). Investigation of serum response to PMP22, connexin 32 and P(0) in inflammatory neuropathies. J. Neuroimmunol..

[8] Makowska A (2008). Immune responses to myelin proteins in Guillain-Barré syndrome. J. Neurol. Neurosurg. Psychiatry.

[9] Khalili-Shirazi A, Atkinson P, Gregson N, Hughes RA (1993). Antibody responses to P0 and P2 myelin proteins in Guillain-Barre syndrome and chronic idiopathic demyelinating polyradiculoneuropathy. J Neuroimmunol.

[10] Inglis HR, Csurhes PA, McCombe PA (2007). Antibody responses to peptides of peripheral nerve myelin proteins P0 and P2 in patients with inflammatory demyelinating neuropathy. J. Neurol. Neurosurg. Psychiatry.

[11] Kuwahara M, Suzuki S, Takada K, Kusunoki S (2011). Antibodies to LM1 and LM1-containing ganglioside complexes in Guillain-Barré syndrome and chronic inflammatory demyelinating polyneuropathy. J. Neuroimmunol..

[12] Kuwahara M (2013). Clinical features of CIDP with LM1-associated antibodies. J. Neurol. Neurosurg. Psychiatry.

[13] Devaux JJ, Odaka M, Yuki N (2012). Nodal proteins are target antigens in Guillain-Barré syndrome. J. Peripher. Nerv. Syst..

[14] Man JK (2012). Neurofascin as a target for autoantibodies in peripheral neuropathies. Neurology.

[15] Querol L (2013). Antibodies to contactin-1 in chronic inflammatory demyelinating polyneuropathy. Ann. Neurol..

[16] Delmont E (2017). Autoantibodies to nodal isoforms of neurofascin in chronic inflammatory demyelinating polyneuropathy. Brain.

[17] Doppler K (2016). Auto-antibodies to contactin-associated protein 1 (Caspr) in two patients with painful inflammatory neuropathy. Brain.

[18] Querol L (2014). Neurofascin IgG4 antibodies in CIDP associate with disabling tremor and poor response to IVIg. Neurology.

[19] Doppler K (2015). Destruction of paranodal architecture in inflammatory neuropathy with anti-contactin-1 autoantibodies. J. Neurol. Neurosurg. Psychiatry.

[20] Ogata H (2015). Characterization of IgG4 anti-neurofascin 155 antibody-positive polyneuropathy. Ann. Clin. Transl. Neurol..

[21] Miura Y (2015). Contactin 1 IgG4 associates to chronic inflammatory demyelinating polyneuropathy with sensory ataxia. Brain.

[22] Devaux JJ (2016). Neurofascin-155 IgG4 in chronic inflammatory demyelinating polyneuropathy. Neurology.

[23] Querol L (2015). Rituximab in treatment-resistant CIDP with antibodies against paranodal proteins. Neurol. Neuroimmunol. Neuroinflammation.

[24] Manso C, Querol L, Mekaouche M, Illa I, Devaux JJ (2016). Contactin-1 IgG4 antibodies cause paranode dismantling and conduction defects. Brain.

[25] Mehndiratta, M. M., Hughes, R. A. C. & Pritchard, J. Plasma exchange for chronic inflammatory demyelinating polyradiculoneuropathy. *Cochrane database Syst. Rev*. CD003906 10.1002/14651858.CD003906.pub4 (2015). 10.1002/14651858.CD003906.pub4 PMC6734114 26305459

[26] Eftimov F, Winer JB, Vermeulen M, de Haan R, van Schaik IN (2013). Intravenous immunoglobulin for chronic inflammatory demyelinating polyradiculoneuropathy. Cochrane database Syst. Rev..

[27] Berger M, McCallus DE, Lin CS-Y (2013). Rapid and reversible responses to IVIG in autoimmune neuromuscular diseases suggest mechanisms of action involving competition with functionally important autoantibodies. J. Peripher. Nerv. Syst..

[28] Dalakas MC, Engel WK (1980). Immunoglobulin and Complement Deposits in Nerves of Patients With Chronic Relapsing Polyneuropathy. Arch Neurol.

[29] Yan WX, Taylor J, Andrias-Kauba S, Pollard JD (2000). Passive transfer of demyelination by serum or IgG from chronic inflammatory demyelinating polyneuropathy patients. Ann. Neurol..

[30] Csurhes P (2005). A, Sullivan, A-A, Green, K., Pender, M. P. & McCombe, P. A. T cell reactivity to P0, P2, PMP-22, and myelin basic protein in patients with Guillain-Barre syndrome and chronic inflammatory demyelinating polyradiculoneuropathy. J. Neurol. Neurosurg. Psychiatry.

[31] Hughes RAC, Allen D, Makowska A, Gregson NA (2006). Pathogenesis of chronic inflammatory demyelinating polyradiculoneuropathy. J. Peripher. Nerv. Syst..

[32] Sanvito L (2009). Humoral and cellular immune responses to myelin protein peptides in chronic inflammatory demyelinating polyradiculoneuropathy. J. Neurol. Neurosurg. Psychiatry.

[33] Willison HJ, Yuki N (2002). Peripheral neuropathies and anti-glycolipid antibodies. Brain.

[34] Ilyas AA, Mithen FA, Dalakas MC, Chen ZW, Cook SD (1992). Antibodies to acidic glycolipids in Guillain-Barré syndrome and chronic inflammatory demyelinating polyneuropathy. J. Neurol. Sci..

[35] van Schaik IN, Vermeulen M, van Doorn PA, Brand A (1994). Anti-GM1 antibodies in patients with chronic inflammatory demyelinating polyneuropathy (CIDP) treated with intravenous immunoglobulin (IVIg). J. Neuroimmunol..

[36] Verschueren A (2005). Lewis-Sumner syndrome and multifocal motor neuropathy. Muscle and Nerve.

[37] Rajabally YA, Chavada G (2009). Lewis-Sumner syndrome of pure upper-limb onset: Diagnostic, prognostic, and therapeutic features. Muscle and Nerve.

[38] Kwa MSG (2003). Autoimmunoreactivity to Schwann cells in patients with inflammatory neuropathies. Brain.

[39] Hernández AM, Rodríguez-Zhurbenko N (2017). Detection of naturally occurring human antibodies against gangliosides by ELISA. Methods Mol. Biol..

[40] Ishibashi T (2004). Tetraspanin protein CD9 is a novel paranodal component regulating paranodal junctional formation. J. Neurosci..

[41] Miyaji K, Paul F, Shahrizaila N, Umapathi T, Yuki N (2016). Autoantibodies to tetraspanins (CD9, CD81 and CD82) in demyelinating diseases. J. Neuroimmunol..

[42] Harschnitz O (2016). Autoantibody pathogenicity in a multifocal motor neuropathy induced pluripotent stem cell-derived model. Ann. Neurol..

[43] Labasque M (2014). Specific contactin N-glycans are implicated in neurofascin binding and autoimmune targeting in peripheral neuropathies. J. Biol. Chem..

[44] Brockes JP, Fields KL, Raff MC (1979). Studies on cultured rat Schwann cells. I. Establishment of purified populations from cultures of peripheral nerve. Brain Res..

[45] Li R (1998). Culture methods for selective growth of normal rat and human Schwann cells. Methods Cell Biol..

[46] Willison HJ (1999). Inter-laboratory validation of an ELISA for the determination of serum anti-ganglioside antibodies. Eur. J. Neurol..

[47] O’Hanlon GM (2000). Peripheral neuropathy associated with anti-GM2 ganglioside antibodies: clinical and immunopathological studies. Autoimmunity.

