# Supplementary material for: Antibodies against peripheral nerve antigens in chronic inflammatory demyelinating polyradiculoneuropathy
Source: Sci Rep. 2017 Oct 31;7:14411. doi: 10.1038/s41598-017-14853-4 (PMC5663697; doi:10.1038/s41598-017-14853-4)
Supplement: Supplementary file 1 — Supplementary Information [file 41598_2017_14853_MOESM1_ESM.pdf]

Antibodies against peripheral nerve antigens in chronic inflammatory demyelinating polyradiculoneuropathy.

Luis Querol* MD PhD[1,2], Ana Mª Siles* MSc[1,2], Roser Alba-Rovira MSc[1,2], Agustín Jáuregui MD[3], Jerome Devaux[4] PhD, Catherine Faivre-Sarrailh[4] PhD, Josefa Araque BN[1,2], Ricard Rojas-Garcia MD PhD[1,2], Jordi Diaz-Manera MD PhD[1,2], Elena Cortés-Vicente MD[1,2], Gisela Nogales-Gadea PhD[5], Miquel Navas-Madroñal MSc[1,2], Eduard Gallardo PhD[1,2], Isabel Illa MD PhD[1,2].

*These authors equally contributed to this work.

1. Neuromuscular Diseases Unit, Department of Neurology, Hospital de la Santa Creu i Sant Pau, Universitat Autònoma de Barcelona, Barcelona, Spain.
2. Centro para la Investigación Biomédica en Red en Enfermedades Raras, CIBERER, Madrid, Spain.
3. Fundación Favaloro, Buenos Aires, Argentina
4. Aix-Marseille Université, CNRS, CRN2M-UMR7286, Marseille, France.
5. Fundació Institut d'Investigació en Ciències de la Salut Germans Trias i Pujol, Badalona, Spain.

PATIENT ID CODE	AGE	GENDER	CIDP SUBTYPE
1	60	M	TYPICAL
2	56	F	TYPICAL
3	63	M	TYPICAL
4	ND	M	TYPICAL
5	30	F	ATYPICAL: PURE ATAXIC
6	92	F	ATYPICAL: LEWIS-SUMNER
7	84	M	TYPICAL
8	55	F	TYPICAL
9	79	F	TYPICAL
10	80	F	TYPICAL
11	39	F	TYPICAL
12	84	M	ATYPICAL: PURE ATAXIC
13	77	F	TYPICAL
14	57	F	TYPICAL
15	62	M	TYPICAL
16	68	M	TYPICAL
17	35	M	TYPICAL
18	60	F	ATYPICAL:DADS
19	37	F	TYPICAL
20	21	M	ATYPICAL:DADS
21	70	M	TYPICAL
22	46	M	TYPICAL
23	69	F	ATYPICAL: PURE ATAXIC
24	69	M	TYPICAL
25	64	F	ATYPICAL: LEWIS-SUMNER
26	75	M	TYPICAL
27	80	F	TYPICAL
28	71	F	TYPICAL
29	62	M	TYPICAL
30	59	M	TYPICAL
31	66	M	ATYPICAL: LEWIS-SUMNER
32	43	M	TYPICAL
33	71	M	ATYPICAL: LEWIS-SUMNER
34	26	M	ATYPICAL:DADS
35	62	F	TYPICAL
36	68	M	TYPICAL
37	84	M	ATYPICAL: PURE SENSORY
38	58	M	TYPICAL
39	66	M	ATYPICAL:DADS
40	73	M	TYPICAL
41	ND	M	TYPICAL
42	ND	M	TYPICAL
43	71	F	TYPICAL
44	82	F	ATYPICAL: PURE SENSORY
45	48	F	TYPICAL
46	74	F	TYPICAL
47	66	M	ATYPICAL: LEWIS-SUMNER
48	53	M	ATYPICAL: LEWIS-SUMNER
49	54	F	TYPICAL
50	74	M	ATYPICAL:DADS
51	81	F	TYPICAL
52	49	F	TYPICAL
53	83	F	ATYPICAL:DADS
54	68	M	TYPICAL
55	66	M	ATYPICAL:DADS
56	76	M	TYPICAL
57	57	F	TYPICAL
58	44	M	ATYPICAL: PURE SENSORY
59	32	F	TYPICAL
60	60	F	ATYPICAL: LEWIS-SUMNER
61	ND	F	TYPICAL
62	92	F	TYPICAL
63	53	M	TYPICAL
64	48	M	ATYPICAL: LEWIS-SUMNER
65	63	M	TYPICAL

Supplementary Table S1. Patients' demographical description. DADS: distal acquired demyelinating symmetric.

PATIENT ID	MOTOR NEURONS IgG	SCHWANN CELLS IgG	SCHWANN CELLS IgM	DRG NEURONS IgG	DRG NEURONS IgM	CNTN1	CNTN1 + CASPR1	NF155	CNTN2+ CASPR2	PMP2	MPZ	Gliomedin	NrCAM	NavB1	NavB2	CD9	L1CAM
1	0	0	0	0	0	0	0	0	0	0	0	0	0	0	0	0	0
2	1	1	0	1	0	0	0	0	0	0	0	0	0	0	0	0	0
3	0	0	0	1	1	0	0	0	0	0	0	0	0	0	0	0	0
4	3	0	0	3	0	3	3	0	0	0	0	0	0	0	0	0	0
5	0	0	0	0	0	0	0	0	0	0	0	0	0	0	0	0	0
6	0	0	0	0	0	0	0	0	0	0	0	0	0	0	0	0	0
7	0	0	0	0	0	0	0	0	0	0	0	0	0	0	0	0	0
8	0	0	0	0	0	0	0	0	0	0	0	0	0	0	0	0	0
9	0	0	0	0	0	0	0	0	0	0	0	0	0	0	0	0	0
10	0	0	0	0	1	0	0	0	0	0	0	0	0	0	0	0	0
11	0	0	0	1	0	0	0	0	0	0	0	0	0	0	0	0	0
12	0	0	0	2	0	0	0	0	0	0	0	0	0	0	0	0	0
13	0	0	0	0	0	0	0	0	0	0	0	0	0	0	0	0	0
14	0	0	0	0	0	0	0	0	0	0	0	0	0	0	0	0	0
15	0	0	0	0	0	0	0	0	0	0	0	0	0	0	0	0	0
16	0	0	0	0	0	0	0	0	0	0	0	0	0	0	0	0	0
17	0	0	0	0	0	0	0	0	0	0	0	0	0	0	0	0	0
18	0	0	0	1	2	0	0	0	0		0						
19	0	1	0	1	0	0	0	0	0	0	0	0	0	0	0	0	0
20	0	0	0	0	0	0	0	3	0		0						
21	0	0	0	1	0	0	0	0	0		0						
22	0	0	0	0	0	0	0	0	0	3	0	0	0	0	0	0	0
23	0	3	1	1	0	0	0	0	0	0	0	0	0	0	0	0	0
24	0	0	0	0	0	0	0	0	0	0	0	0	0	0	0	0	0
25	0	0	0	0	0	0	0	0	0	0	0	0	0		0	0	0
26	3	0	1	3	0	3	3	0	0	0	0	0		0	0	0	0
27	0	0	0	0	0	0	0	0	0	0	0	0	0	0	0	0	0
28	0	0	0	1	1	0	0	0	0	0	0	0	0	0	0	0	0
29	0	0	0	0	0	0	0	0	0	0	0	0	0	0	0	0	0
30	1	0	0	0	1	0	0	0	0	0	0	0	0	0	0	0	0
31	0	0	0	0	0	0	0	0	0	0	0	0	0	0	0	0	0
32	0	1	0	0	0	0	0	0	0	0	0	0	0	0	0	0	0
33	0	0		3		0	0	0		0	0	0	0	0	0		0
34	0	0	0	0	0	0	0	3	0	0	0	0	0	0	0		0
35	0	0	0	0	0	0	0	0	0	0	0	0	0	0	0	0	0
36	0	0	0	1	0	0	0	0	0	0	0	0	0	0	0	0	0
37	0	0	0	0	0	0	0	0	0	0	0	0	0	0	0	0	0
38	0	0	0	0	0	0	0	0	0	0	0	0	0	0	0	0	0
39	0	0	0	0	0	0	0	3	0	0	0	0	0	0	0	0	0
40	0	0	0	0	0	0	0	0	0	0	0	0	0	0	0	0	0
41	1	0	0	0	0	0	0	0	0	0	0	0	0	0	0	0	0
42	3	1	0	2	0	3	3	0	0	0	0	0	0	0	0	0	0
43	0	0	0	0	0	0	0	0	0	0	0	0	0	0	0	0	0
44	0	0	0	0	0	0	0	0	0	0	0	0	0	0	0	0	0
45	0	0	0	0	0	0	0	0	0	0	0	0	0	0	0	0	0
46	0	0	0	0	0	0	0	0	0	0	0	0	0	0	0	0	0
47	0	0	0	0	0	0	0	0	0	0	0	0	0	0	0	0	0
48	0	0	0	2	0	0	0	0	0	0	0	0	0	0	0	0	0
49	0	0	0	0	0	0	0	0	0	0	0	0	0	0	0	0	0
50	0	3	1	0	0	0	0	0	0	0	0	0	0	0	0	0	0
51	0	2	0	3	0	0	3	0	0	0	0	0	0	0	0	0	0
52	0	0	0	0	0	0	0	0	0	0	0	0	0	0	0	0	0
53	0	0	0	1	0	0	0	0	0	0	0	0	0	0	0	0	0
54	0	0	0	0	0	0	0	0	0	0	0	0	0	0	0	0	0
55	0	0	0	0	0	0	0	0	0	0	0	0	0	0	0	0	0
56	0	2	0	0	0	0	0	0	0	0	0	0	0	0	0	0	0
57	0	0	0	0	0	0	0	0	0	0	0	0	0	0	0	0	0
58	0	0	2	0	0	0	0	0	0	0	0	0	0	0	0	0	0
59	0	0	0	0	0	0	0	0	0	0	0	0	0	0	0	0	0
60	0	0	0	0	1	0	0	0	0	0	0	0	0	0	0	0	0
61	0	0	0	0	0	0	0	0	0	0	0	0	0		0	0	0
62	3	2	0	3	0	3	3	0	0	0	0	0	0	0	0	0	0
63	0	0	0	0	0	0	0	0	0	0	0	0	0	0	0	0	0
64	0	1	0	2	0	0	0	0	0	0	0	0	0	0	0	0	0
65	0	0	0	0	0	0	0	0	0	0	0	0	0	0	0	0	0

Supplementary Table S2. All ICC results.

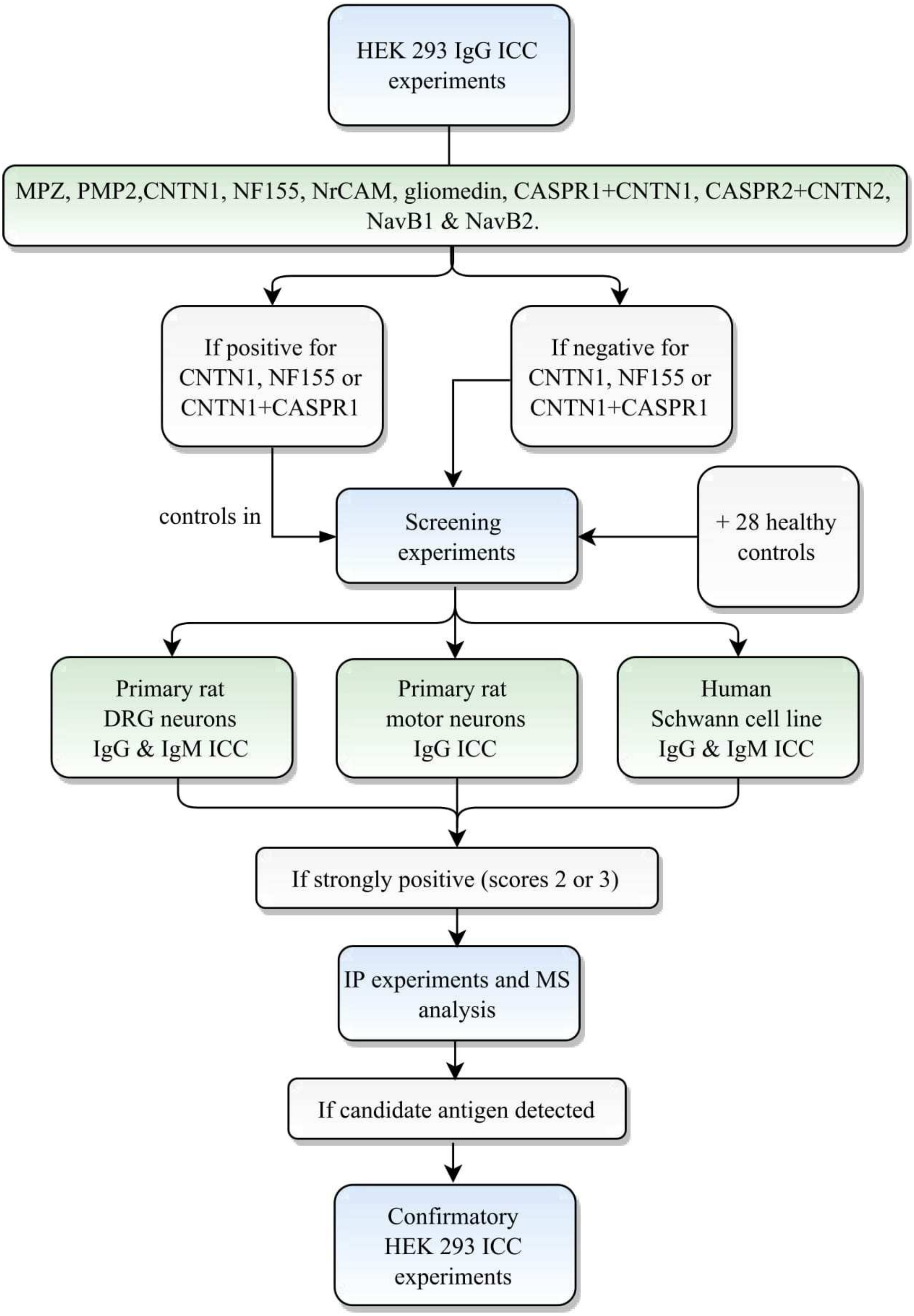

Supplementary Figure S1. Autoantibody screening protocol diagram. MS: Mass spectrometry.

cDNA Clone	Protein of interest	Permeabilization	Sera dilution	Blocking solution	Commercial antibody	Commercial antibody dilution	Secondary antibodies	Secondary antibodies dilution
pCMV6-NFASC-Myc-DDK RC228652 (Origene)	**NF155**	NO	1/100	Goat 5% in PBS	Chicken NF (AF3235 R&D systems)	1/1000	GAC488 (A11039 Molecular Probes) + GAH594 (Ab98621 Abcam)	1/1000+1/1000
pCMV6-CNTN1-untagged EX-A1153-M02 (Genecopoeia)	**CNTN-1**	NO	1/100	Rabbit 1/40 in PBS	Goat CNTN1 (AF904 R&D systems)	1/1000	RAG488 (A11078 Thermo Scientific) + RAH594 (SA510112 Thermo Scientific)	1/1000+1/1000
pCMV6-CNTN1-untagged EX-A1153-M02 (Genecopoeia) + pCMV6-CNTNAP1-untagged EX-M0417-M02 (Genecopoeia)	**CNTN-1+CASPR-1**	NO	1/100	Rabbit 1/40 in PBS	Goat CNTN1 (AF904 R&D systems)	1/1000	RAG488 (A11078 Thermo Scientific) + RAH594 (SA510112 Thermo Scientific)	1/1000+1/1000
pCMV6-CNTN2-untagged EX-A1282-M02B (Genecopoeia) + CASPR2 vector kindly provided by Dr. Graus	**CNTN-2+CASPR-2**	NO	1/100	Goat 5% in PBS	Rabbit CNTN2 (AB133498 Abcam)	1/200	GAR 488 (A11008 Thermo Scientific) + GAH594 (Ab98621 Abcam)	1/500 +1/500
in-house MPZ ORF cloned in a pcDNA40 vector (Genecopoeia)	**MPZ**	YES	1/100	Goat 5% in PBS	Chicken MPZ (LS-C149138LifeSpan BioSciences)	1/200	GAC488 (A11039 Molecular Probes) + GAH594 (Ab98621 Abcam)	1/500 +1/500
in-house PMP2 ORF cloned in a pcDNA40 vector (Genecopoeia)	**PMP2**	YES	1/40	Goat 5% in PBS	Rabbit anti-PMP2 (12717-1-AP ProteinTech Group)	1/200	GAR 594 (A11037 Thermo Scientific) + GAH488 (A11013 Thermo Scientific)	1/500 +1/500
pIRES-NrCAM-HA courtesy of Drs. Devaux and Faivre-Sarrailh	**NrCAM**	YES	1/40	Goat 5% in PBS	Rat-anti-HA (11-867-423-001 Roche)	1/2000	GARat 488 (A11006 Thermo Scientific) + GAH594 (Ab98621 Abcam)	1/500 +1/500
pcDNA3-Gliomedin-Myc courtesy of Drs. Devaux and Faivre-Sarrailh	**gliomedin**	YES	1/40	Goat 5% in PBS	Mouse-anti-Myc (CBL430 Millipore)	1/200	GAM 488 (A-11001 Molecular Probes) + GAH594 (Ab98621 Abcam)	1/500 +1/500
pcDNA3-NavB1-HA courtesy of Drs. Devaux and Faivre-Sarrailh	**NavB1**	YES	1/40	Goat 5% in PBS	Rat-anti-HA (11-867-423-001 Roche)	1/2000	GARat 488 (A11006 Thermo Scientific) + GAH594 (Ab98621 Abcam)	1/500 +1/500
pcDNA3-NavB2-Myc courtesy of Drs. Devaux and Faivre-Sarrailh	**NavB2**	NO	1/40	Goat 5% in PBS	Mouse-anti-Myc (CBL430 Millipore)	1/200	GAM 488 (A-11001 Molecular Probes) + GAH594 (Ab98621 Abcam)	1/500 +1/500
pCMV6-CD9-Myc-DDK RC202000 (Origene)	**CD9**	NO	1/40	Goat 5% in PBS	Mouse-anti-Myc (CBL430 Millipore)	1/200	GAM 488 (A-11001 Molecular Probes) + GAH594 (Ab98621 Abcam)	1/500 +1/500
pCMV6-L1CAM -Myc-DDK RC211601 (Origene)	**L1CAM**	YES	1/40	Goat 5% in PBS	Mouse-anti-Myc (CBL430 Millipore)	1/200	GAM 488 (A-11001 Molecular Probes) + GAH594 (Ab98621 Abcam)	1/500 +1/500

Supplementary Table S3. HEK293 cell transfection and ICC conditions.
